# Urinary oxytocin positively correlates with performance in facial visual search in unmarried males, without specific reaction to infant face

**DOI:** 10.3389/fnins.2014.00217

**Published:** 2014-07-29

**Authors:** Atsuko Saito, Hiroki Hamada, Takefumi Kikusui, Kazutaka Mogi, Miho Nagasawa, Shohei Mitsui, Takashi Higuchi, Toshikazu Hasegawa, Kazuo Hiraki

**Affiliations:** ^1^Department of Cognitive and Behavioral Science, Graduate School of Arts and Sciences, The University of TokyoTokyo, Japan; ^2^Department of General Systems Studies, Graduate School of Arts and Sciences, The University of TokyoTokyo, Japan; ^3^Department of Animal Science and Biotechnology, Azabu UniversityKanagawa, Japan; ^4^Faculty of Medical Sciences, University of FukuiFukui, Japan; ^5^JST, CRESTTokyo, Japan

**Keywords:** oxytocin, social cognition, infant, visual search, cognitive task

## Abstract

The neuropeptide oxytocin plays a central role in prosocial and parental behavior in non-human mammals as well as humans. It has been suggested that oxytocin may affect visual processing of infant faces and emotional reaction to infants. Healthy male volunteers (*N* = 13) were tested for their ability to detect infant or adult faces among adult or infant faces (facial visual search task). Urine samples were collected from all participants before the study to measure the concentration of oxytocin. Urinary oxytocin positively correlated with performance in the facial visual search task. However, task performance and its correlation with oxytocin concentration did not differ between infant faces and adult faces. Our data suggests that endogenous oxytocin is related to facial visual cognition, but does not promote infant-specific responses in unmarried men who are not fathers.

## Introduction

Oxytocin is a 9-amino-acid peptide that is produced in the hypothalamus and is released into the brain and bloodstream (Donaldson and Young, 2008). It was originally known as a hormone that increases uterus contractions during labor and stimulates the ejection of milk. In the past few decades, although controversial, current evidence suggest that oxytocin has a role to play in social and maternal behavior. Rats treated with oxytocin receptor antagonists and oxytocin knockout mice are unable to recognize previously encountered conspecifics (Engelmann et al., 1998; Ferguson et al., 2000). In prairie voles, infusion of oxytocin in the ventricle promotes pair bond formation in virgin females (Williams et al., 1994; Insel and Hulihan, 1995). Infusion of oxytocin into the ventricle can also initiate maternal behaviors in virgin female rats, such as nest building as well as licking and retrieving pups (Pedersen and Prange, 1979; Pedersen et al., 1982).

In humans, the role of oxytocin in maternal behavior and mother–infant relations, as well as in paternal behavior and father–infant relations, has been investigated. Plasma oxytocin levels in mothers positively correlated to the amount of maternal bonding behaviors, such as gaze, vocalizations, affectionate touch, mother–infant behavioral synchrony, and attachment style (Feldman et al., 2007, 2011; Strathearn et al., 2009; Gordon et al., 2010a,b). Paternal behavior and father–infant affect synchrony have also been associated with oxytocin levels in plasma and saliva (Gordon et al., 2010a,b,c; Feldman et al., 2011). Both fathers and mothers who provide high levels of contact toward their infants show increased salivary oxytocin following parent–infant interactions, and such an increase is not observed among parents displaying low levels of contact (Feldman et al., 2010). These results suggest that peripheral oxytocin levels are positively related to maternal and paternal behavior, and to parental attachment to infants in mothers and fathers.

Evidence indicating that intranasal oxytocin administration affects social perception, cognition, and behavior in a non-parental context is accumulating (Bos et al., 2012; Churchland and Winkielman, 2012; Guastella and Macleod, 2012; Van Ijzendoorn and Bakermans-Kranenburg, 2012; Zink and Meyer-Lindenberg, 2012). However, it is still not clear whether exogenously administered oxytocin, which increases oxytocin level temporally, has the same effects on social cognition or behavior as basal oxytocin levels. In fact, in the context of a trust game, administered oxytocin causes a substantial increase in trust (Kosfeld et al., 2005), but baseline plasma oxytocin levels do not associate with trust (Zak et al., 2005). In parental contexts, although some research investigated the relationship between oxytocin and perception for infants stimuli or paternal behavior by using intranasal oxytocin administration (Riem et al., 2011; Rupp et al., 2012; Weisman et al., 2012, 2014), the role of oxytocin in infant stimuli perception remains to be evaluated (Van Ijzendoorn and Bakermans-Kranenburg, 2012).

The cognitive processes associated with the perception of infant stimuli that relate to parental behavior are unknown. As mentioned above, many previous studies investigating the relationship between parental behavior and endocrinology focused on observed, direct behavior toward infants, or subjective evaluation of emotion against infants in parents (Fleming et al., 1987; Feldman et al., 2007; Gordon et al., 2010a). In addition, some neuroimaging studies have examined brain activation in response to infant stimuli (Swain, 2008), and a few researchers have reported the cognitive effects of infant stimuli (Brosch et al., 2007, 2008; Nittono et al., 2012). However, the question of how infant stimuli are processed, cognitively, remains open. Given the response to infant stimuli can be related to maltreatment of infants, it is important for researchers to understand these cognitive processes.

In rodents, the role of endocrinological factors, including oxytocin, in parental behavior has been investigated in both parental and non-parental individuals. However, in humans, it is mainly investigated in parents. Currently, we do not know whether oxytocin has the same role in non-parental humans as observed in parental individuals because parents have a different physiological status from those of non-parents. For example, fathers and mothers who have young children have lower testosterone levels than non-fathers and non-mothers (Kuzawa et al., 2010; Gettler et al., 2011). Compared to non-fathers, primate fathers that help mothers raise their young like humans have an increased density of dendritic spines on pyramidal neurons and increased vasopressin receptors in the prefrontal cortex (Kozorovitskiy et al., 2006). Therefore, the effect of oxytocin on parental behavior or response to infant stimuli in non-parents may differ from that in parents.

The purpose of this study was to investigate the role of oxytocin in non-parents in response to infant faces with comparison to adult faces. We measured urinary oxytocin levels just before the cognitive test in non-married, non-father men. We adopted the baseline peripheral oxytocin levels because nearly all previous research about oxytocin's role in parental behavior has treated them as mentioned above. We measured urinary oxytocin because it is related to social interactions (Fries et al., 2005; Nagasawa et al., 2009; Seltzer et al., 2010; Snowdon et al., 2010; Crockford et al., 2013; Wittig et al., 2014) and its sampling is non-invasive. Urinary oxytocin has a linear association with plasma oxytocin level (Amico et al., 1987; Romero et al., 2014). Women were excluded from our study to rule out possible interactions with circulating gonadal steroids (Salonia et al., 2005). Because infant faces attract human attention in the dot-probe task (Brosch et al., 2007, 2008), we measured non-parents' reaction to infants by using the attention task. The task was visual search of infant and adult faces. This task is important, requiring attention during an active scan of the visual environment, as participants search for a particular object or feature (the target) among other objects or features (distracters); it is considered a key paradigm in attention research for the investigation of selective attention (Muller and Krummenacher, 2006). If oxytocin is positively related to the responsiveness to infants, the performance for infant faces, or the performance difference between the reaction time for infant faces and that for adult faces, will negatively correlate with urinary oxytocin levels.

## Materials and methods

### Participants

Thirteen healthy young men aged 21–33 years (*M* = 26.08, *SD* = 3.43) were recruited from the student population of the University of Tokyo. All had normal or corrected-to-normal vision according to self-report. All were non-married and had no offspring. All protocols were approved by the Research Ethics Committee of the University of Tokyo (subject No. 229-2). Written informed consent was obtained from the participants and participants gave permission to use their data in the analyses.

### Stimuli and apparatus

The stimuli consisted of adult stimuli and infant stimuli. Twenty chromatic infant face photographs consisting of 10 male and 10 female Japanese infants aged from 6 to 9 months old (*M* = 6.5, *SD* = 0.81) were prepared to make infant stimuli. Ten chromatic adult face photographs consisting of 6 male and 4 female Japanese adults aged 19–34 years (*M* = 23.7, *SD* = 4.43) and 10 photographs from the Ekman and Friesen (1976) database consisting of 4 male and 6 female Asian neutral faces were prepared to make adult stimuli. These 40 photographs were morphed using Sqirlz Morph software (Xiberpix, Solihull, UK; http://www.xiberpix.net/SqirlzMorph.html). First, 4 morphed faces were made from the following categories: adult males, adult females, infant males, and infant females. These 4 morphed faces were mixed with each original face of the same category with a mixture ratio of 1:2. All photographs were presented in gray scale, matched in brightness and contrast, and pasted onto a black background.

A 16-inch color CRT monitor attached to a PC was used to display the experimental tasks. The experimental control software was written with E-Prime 2.0 (Psychological Software Tools).

### Procedure

**Figure 1:**
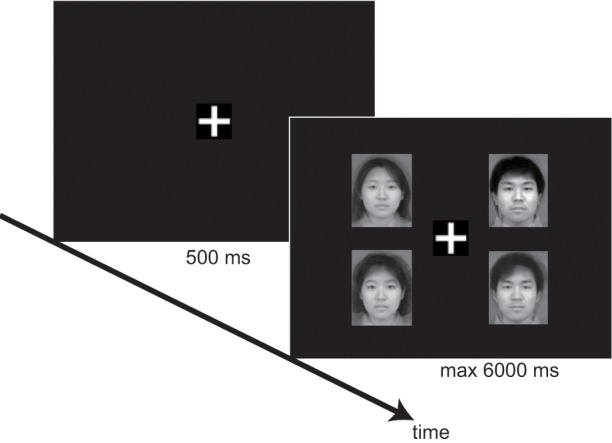
**Experimental sequence of the visual search task**.

Performances were tested using a within-subjects design with 3 factors: target (present vs. absent), distracter's age category (adult vs. infant), and set size—the number of elements in the presentation. We used a variety of set sizes: 3, 4, and 6. Each trial started with the presentation of a white fixation cross (1.2 × 1.2 cm) for 500 ms. Next, the stimulus faces were presented. The faces were 5.7 cm high × 4.5 cm wide on the screen, and were presented at a distance of 5.8 cm between the fixation cross and the center of the image. Participants were placed 60 cm from the screen using a chin-rest. This resulted in a visual angle of 5.5° between the fixation cross and the center of the image. Participants were instructed to press the “same” button (a numeric key 1 or 2) if all faces were the same age category and the “different” button (a numeric key 2 or 1) if one of the faces differed in the age category from the rest. The combinations of numeric keys were assigned randomly to each participant. Participants were also asked to respond as quickly and accurately as possible. The stimulus faces remained on the screen for 6000 ms or until the participant responded (Figure 1).

For each set size, there were 160 trials composed of 4 blocks of 40. There were 12 blocks in total, which were preceded by a block of 8 practice trials. The order of the blocks differed across participants.

### Measurement of urinary oxytocin

Urine was collected from participants just before the visual search task between 1400 and 1800. Participants were instructed to abstain from food and drink for 2 h before urine sampling. An hour before this urine sampling, participants urinated and were instructed to abstain from exercise, stressful activity (e.g., seeing exciting movies), and sleeping. Immediately after collection, urine samples were centrifuged at 4°C in a refrigerated centrifuge, and frozen at −80°C until assayed. Urinary oxytocin concentrations were measured using radioimmunoassays (Mitsui et al., 2011). Creatinine concentrations were measured using the Jaffe reaction using 96-well microplates (3881-096, Asahi Glass Co., Japan). The plate was read at an optical density of 450 nm by using a microplate reader. Urinary oxytocin levels are expressed as the oxytocin to creatinine ratio. The intra-assay coefficient of variation was 4.26%.

## Results

### Behavioral data

Data from all participants were analyzed because error rates were lower than 10%. The average error rate was 5.0%. Only reaction times (RTs) to correctly identified targets and correctly rejected non-targets were included in the analyses. Before the analysis, RTs were filtered for outliers. All RTs lying more than 2 standard deviations above or below the individual mean were excluded from the analyses. Average RTs and differences between the reaction time for infant faces and for adult faces (RTs for Infant target or distracters - RTs for Adult target or distracters) are summarized in Table 1.

**Table 1:** **Average RTs (ms) and standard deviations for target-present and target-absent trials and differences between the reaction time for infant faces and that for adult faces**.

**Set size**	**3**		**4**		**6**	
	***M***	***SD***	***M***	***SD***	***M***	***SD***
**TARGET PRESENT**						
Adult target	855.259	155.215	873.335	117.583	972.834	123.608
Infant target	843.201	135.803	875.097	137.499	963.807	90.290
Difference	–12.059	50.304	1.762	70.636	–9.027	65.710
**TARGET ABSENT**						
Adult all	856.670	149.689	860.864	120.309	1046.755	138.065
Infant all	742.284	102.438	752.219	101.178	937.447	105.662
Difference	–114.386	68.782	–108.645	64.621	–109.308	86.976

### Association between oxytocin levels and performance in visual search

**Figure 2:**
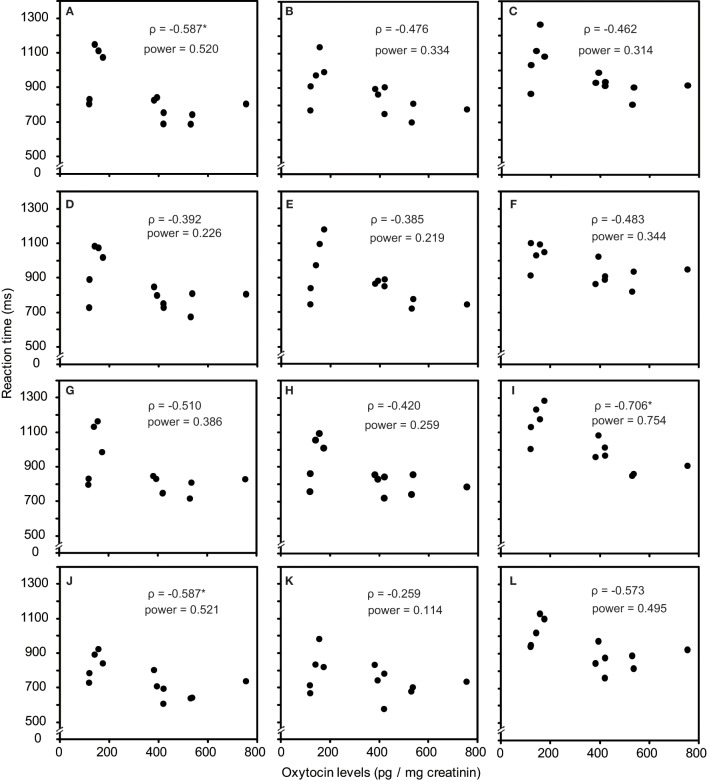
**Correlation between urinary oxytocin levels and reaction times (A–L). (A)** Target present, adult target condition, set size 3; **(B)** Target present, adult target condition, set size 4; **(C)** Target present, adult target condition, set size 6; **(D)** Target present, infant target condition, set size 3; **(E)** Target present, infant target condition, set size 4; **(F)** Target present, infant target condition, set size 6; **(G)** Target absent, adult all condition, set size 3; **(H)** Target absent, adult all condition, set size 4; **(I)** Target absent, adult all condition, set size 6; **(J)** Target absent, infant all condition, set size 3; **(K)** Target absent, infant all condition, set size 4; **(L)** Target absent, infant all condition, set size 6. ^*^indicates *p* < 0.05.

**Figure 3:**
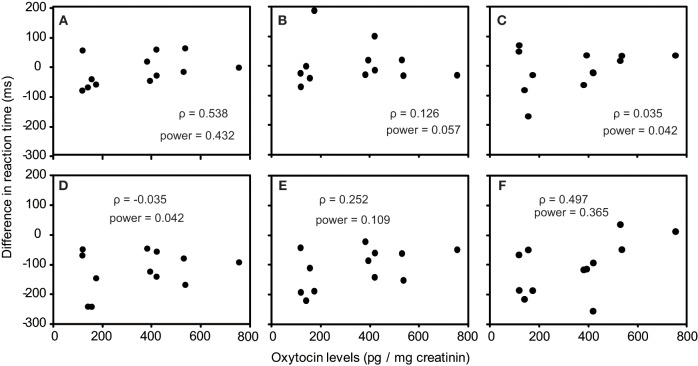
**Correlation between urinary oxytocin levels and differences between the reaction times for infant faces and adult faces (A–F)**. **(A)** Target present, set size 3; **(B)** Target present, set size 4; **(C)** Target present, set size 6; **(D)** Target absent, set size 3; **(E)** Target present, set size 4; **(F)** Target present, set size 6.

As one participant's oxytocin concentration was extremely high (2031.79 pg/mg), we analyzed other 12 participants' data. The average oxytocin concentrations was 344.38 pg/mg (range: 117.38–753.36, *SD* = 196.18). We calculated Speaman's correlation coefficients between RTs in the visual search task and urinary oxytocin levels (Figure 2). As shown in Figures 2A–L, the reaction time for the visual search task negatively correlated with urinary oxytocin levels; that is, the performance positively correlated with oxytocin levels. The correlation coefficients (Speamans rho) were significant for the adult target conditions of set size 3 (*rho* = −0.587, *p* = 0.045, Figure 2A), and the adult condition of set size 6 and the infant condition of set size 3 in target absent condition (*rho* = −0.706, *p* = 0.010; *rho* = −0.587, *p* = 0.045, respectively, Figures 2I,J). We also calculated the statistical power of our experiments. This analysis revealed that correlations that were statistically significant had sufficient statistical power (~0.5, Figure 2). In contrast, there were no consistent tendencies in the differences between the reaction time for infant faces and adult faces (Figures 3A–F).

## Discussion

Our results show that performance of the visual search task positively correlated with urinary oxytocin levels. This is the first presentation of the relationship between peripheral oxytocin levels and the performance of cognitive tasks. The response for social stimuli, both of infant and adult faces, was accelerated by high oxytocin levels. This result is consistent with many previous studies in which intranasal oxytocin administration promoted positive responses in social contexts (Kosfeld et al., 2005; Zak et al., 2007; Baumgartner et al., 2008; Petrovic et al., 2008; Theodoridou et al., 2009). However, arginine vasopressin, a neuropeptide that has a similar amino acid sequence and function as oxytocin, enhanced performance in a simple reaction time task when it was delivered by intranasal spray (Beckwith et al., 1983; Jennings et al., 1986). Oxytocin may also have a similar effect. In this case, the current results can easily be explained by the enhancement effect of oxytocin on the simple reaction time task. To clarify this issue, it is necessary to perform experiments using non-social stimuli. Collectively, although the results are preliminary due to the relatively small sample size, this is the first presentation of the relationship between endogenous oxytocin levels and the performance of cognitive tasks, which can explain the individual differences in visual search ability for social object. But we cannot find any relationship between endogenous oxytocin and visual search for infant faces in unmarried males, indicating that experience of parenting is important for enhance the visual search ability for infants.

We measured oxytocin levels in urine. There is some controversy surrounding the relationship between oxytocin levels in urine and plasma (Amico et al., 1987; Feldman et al., 2011). However, it is understandable that Feldman et al. (2011) described no correlation between urinary oxytocin and plasma oxytocin, as they measured urine and plasma at a single time point. Urinary oxytocin reflects oxytocin that has accumulated in the kidney over a period of approximately an hour. In our study, participants urinated an hour before urine sampling, and were then instructed to abstain from exercise, stressful activities (e.g., watching exciting movies), and sleeping. Therefore, our samples reflect accumulated oxytocin, excreted from the kidney over the course of an hour. In addition, we recently demonstrated a significant positive correlation between plasma oxytocin and urinary oxytocin levels in dogs (Romero et al., 2014). These results indicate that urinary oxytocin is a non-invasive biomarker that can be used to assess oxytocin activity. There is still debate over whether peripheral measures of oxytocin are related to central measures of oxytocin. However, non-invasive measures, such as urinary oxytocin, hold research and therapeutic advantages (Crockford et al., 2014). Our results improve the understanding of effects of peripheral oxytocin.

The results were somewhat different from the expected ones. The oxytocin levels positively correlated to the detected speed of infant faces as well as to that for adult faces. In addition, oxytocin levels did not relate to differences between the reaction time for infant faces and that for adult faces. A positive relationship between peripheral oxytocin and specific reaction to infants was not observed. These results seem inconsistent with the previous results showing that peripheral oxytocin levels positively correlated with paternal behavior (Gordon et al., 2010a,b,c; Feldman et al., 2011). The reason for this inconsistency may be caused by the difference in features of the participants. Almost all of the previous studies investigating the effect of oxytocin on parental behavior targeted actual parents. In contrast, the participants in our studies were non-married and non-father males. Although fathers do not have different peripheral oxytocin levels from non-fathers (Gray et al., 2007), the physical conditions of fathers, including testosterone levels, differ from those of non-fathers (Kuzawa et al., 2010; Gettler et al., 2011). These differences may explain the inconsistency between the previous studies and our study. There is the possibility that other hormonal levels interact with oxytocin and affect parent-like behavior. For example, testosterone affects expression of the oxytocin receptor in central nervous system (Arsenijevic and Tribollet, 1998) and oxytocin interacts with testosterone level and parental behavior (Weisman et al., 2014). That is, people who have different attributions, including marital status or parenthood, have different effects of oxytocin on parental behavior. Becoming fathers may change the response to infants: the effect of oxytocin on responses specialized for infants will be shown in fathers.

Our results reveal that high oxytocin levels do not always facilitate the specific reaction to infants. As Bartz et al. (2011) pointed out, the effects of oxytocin are constrained by situations and/or individualities. Therefore, it is necessary to study them by considering the features of the participants as discussed above. In addition to the different effects of oxytocin depending on the individual traits, a couple of recent studies demonstrated that oxytocin's social effects are context-dependent (Cardoso et al., 2013; Scheele et al., 2014). We should also take the difference in context into consideration when we investigate the effects of oxytocin.

## Author contributions

Conceived and designed the experiments: Atsuko Saito, Hiroki Hamada, and Kazuo Hiraki. Performed the experiments: Hiroki Hamada Analyzed the data: Hiroki Hamada, Atsuko Saito, and Kazuo Hiraki. Hormonal measurement: Hiroki Hamada, Takefumi Kikusui, Kazutaka Mogi, Miho Nagasawa, and Shohei Mitsui and Takashi Higuchi. Prepared the manuscript: Atsuko Saito and Hiroki Hamada. Organized the research project: Kazuo Hiraki and Toshikazu Hasegawa.

### Conflict of interest statement

The authors declare that the research was conducted in the absence of any commercial or financial relationships that could be construed as a potential conflict of interest.
